# Advances in the Research of Nasopharyngeal Carcinoma: A Perspective

**DOI:** 10.1002/cai2.70074

**Published:** 2026-08-17

**Authors:** Zishan Liang, Xian‐Yue Zeng, Jia‐Wei Lv, Guan‐Qun Zhou, Ying Sun, Li Lin

**Affiliations:** ^1^ Department of Radiation Oncology Sun Yat‐sen University Cancer Center Guangzhou China; ^2^ State Key Laboratory of Oncology in South China Guangzhou China; ^3^ Collaborative Innovation Center for Cancer Medicine Guangzhou China; ^4^ Guangdong Key Laboratory of Nasopharyngeal Carcinoma Diagnosis and Therapy Guangzhou China; ^5^ Zhongshan School of Medicine Sun Yat‐sen University Guangzhou China

**Keywords:** antibody–drug conjugate, immunotherapy, nasopharyngeal carcinoma, precision radiotherapy

## Abstract

The year 2025 marked significant advances in precision medicine for nasopharyngeal carcinoma (NPC). This review summarizes recent advances in treatment strategies aimed at addressing longstanding challenges in NPC management, including treatment‐related toxicity, therapeutic resistance, and the limited precision of risk stratification. For locoregionally advanced NPC, a novel efficacy‐enhancing and toxicity‐minimizing strategy has emerged: toxicity minimizing involves replacing toxic concurrent cisplatin chemotherapy with PD‐1 blockade, achieving superior safety while maintaining non‐inferior 3‐year failure‐free survival; meanwhile efficacy‐enhancing employs adjuvant immunotherapy to improve survival specifically in high‐risk patients, supporting the transition toward risk‐adapted strategies. In radiotherapy, international consensus guidelines recommend reducing target volumes and sparing normal tissues to minimize side effects. For recurrent or metastatic NPC, antibody–drug conjugates(ADCs) and novel immunotherapeutic combinations show promise in overcoming treatment resistance and expanding therapeutic options. Furthermore, advances in artificial intelligence and deeper understanding of resistance mechanisms are accelerating clinical innovation. Collectively, these advances underscore the ongoing shift toward reduced toxicity, improved efficacy, and greater precision in the management of NPC.

## Graphical abstract

The year 2025 marks a paradigm shift in nasopharyngeal carcinoma management towards greater precision, reduced toxicity, and enhanced intelligence. This review highlights the key breakthroughs from 2025 in clinical trials, technological innovations, and basic research for NPC, offering insights for clinical practice and future investigations.

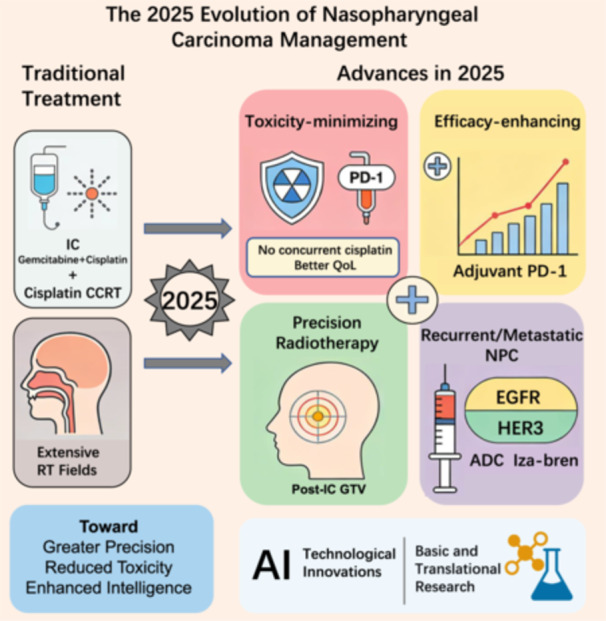

**Abbreviations**
ADC: antibody–drug conjugate
AI: artificial intelligence
CCRT: concurrent chemoradiotherapy
CT: computed tomography
CTV: clinical target volumes
EBV DNA: Epstein–Barr virus deoxyribonucleic acid
EFS: event‐free survival
FFS: failure‐free survival
GP: gemcitabine and cisplatin
GTV: gross tumor volumes
HR: hazard ratio
IC: induction chemotherapy
Iza‐bren: izalontamab brengitecan
LANPC: locoregionally advanced nasopharyngeal carcinoma
LLMs: large language models
MRI: magnetic resonance imaging
NPC: nasopharyngeal carcinoma
ORR: objective response rate
OS: overall survival
QoL: quality of life
RCT: randomized controlled trial
RT: radiotherapy
R/M NPC: recurrent or metastatic nasopharyngeal carcinoma

## 1 Background

Nasopharyngeal carcinoma (NPC) is a head and neck malignancy with unique epidemiological patterns and biological behavior, with a high prevalence in Southern China and Southeast Asia. Over the past two decades, progress in radiotherapy (RT) techniques and combination chemotherapy has significantly improved the survival rates of patients with locoregionally advanced NPC (LANPC), including the widespread use of intensity‐modulated radiotherapy (IMRT) and the combination of induction chemotherapy (IC) with concurrent chemoradiotherapy (CCRT) [1]. However, this intensive and standardized approach faces a twofold challenge. Both extensive irradiation fields and intensive chemotherapy frequently led to severe acute and long‐term toxicities (e.g., xerostomia and dysphagia), which consequently impair the quality of life (QoL) of long‐term survivors [2]. However, approximately 20%–30% of patients still experience disease relapse despite definitive chemoradiotherapy [3]. Consequently, the core challenge lies in reducing toxicity while enhancing efficacy without compromising therapeutic outcomes and identifying novel therapies for high‐risk LANPC and recurrent or metastatic NPC.

Given the evidence from landmark clinical trials and international consensus, the year 2025 marked great advancements in NPC treatment, characterized by the evolution from traditional staging to a precision model defined by risk stratification and molecular biomarkers. Simultaneously, progress has continued in artificial intelligence (AI) technology and the translation of basic research. This review highlights the key breakthroughs from 2025 in clinical trials, technological innovations, and basic research on NPC, offering insights for clinical practice and future investigations.

## 2 Strategies for Enhancing Efficacy and Minimizing Toxicity in Locoregionally Advanced Nasopharyngeal Carcinoma

Landmark studies published in 2025 propose “efficacy‐enhancing and toxicity‐minimizing” strategies to reduce cisplatin toxicity and improve outcomes in high‐risk LANPC, potentially reshaping the treatment landscape (Table 1).

**Table 1:** Summary of landmark trials investigating PD‐1 antibody in locoregionally advanced nasopharyngeal carcinoma.

Strategy	Trial name	Trial ID	Target population (LANPC)	Trial design	Anti–PD‐1 antibody	Intervention period	Sample size	Median follow‐up (months)	Primary endpoint outcome (3‐year Survival)	Safety
Toxicity‐minimizing	DIAMOND	NCT04907370	T4N1M0 or T1‐4N2‐3M0	Randomized, Open‐label, Phase 3 Clinical Trial	Toripalimab	Induction + RT (No Cisplatin) + Adjuvant	532	37.0	FFS: 88.3% (Exp) versus 87.6% (Ctrl) (*p* = 0.002 for noninferiority)	All‐grade vomiting: 26.2% (Exp) versus 59.8% (Ctrl)
	PLATINUM	NCT03984357	T4N1M0 or T1‐4N2‐3M0	Single‐arm, Phase 2 Trial	Nivolumab	Induction + RT (No Cisplatin) + Adjuvant	152	43.0	FFS: 88.5%	Acute Grade 3–4 AEs: 40.2%.
Efficacy‐enhancing	CONTINUUM[6]	NCT03700476	Stage III‐IVa, except T3‐4N0/T3N1	Randomized, Open‐label, Parallel‐group, Phase 3 Clinical Trial	Sintilimab	Induction + Concurrent + Adjuvant	425	41.9	EFS: 86% (Exp) versus 76% (Ctrl)	Acute Grade 3–4 AEs: 74% (Exp) versus 65% (Ctrl).
	DIPPER	NCT03427827	T4N1M0 or T1‐4N2‐3M0	Randomized, Open‐label, Phase 3 Clinical trial	Camrelizumab	Adjuvant	450	39.0	EFS: 86.9% (Exp) versus 77.3% (Ctrl)	Grade 3–4 AEs: 11.2% (Exp) versus 3.2% (Ctrl).

Abbreviations: AE, adverse event; EFS, event‐free survival; FFS, failure‐free survival; RT, radiotherapy; QoL, quality of life.

### 2.1 Toxicity‐Minimizing: Replacing Concurrent Cisplatin Chemotherapy With Immunotherapy

Cisplatin‐based CCRT remains the cornerstone of LANPC treatment. However, its severe toxicity often compromises treatment compliance, thereby affecting not only therapeutic efficacy but also patients’ QoL due to the long‐term sequelae induced by cisplatin. Therefore, mitigating cisplatin chemotherapy toxicity remains a key clinical challenge.

A phase 3 randomized controlled trial (RCT) (*n* = 532) published by The DIAMOND study group was the first to validate the omission of concurrent chemotherapy in a large cohort [4]. This trial compared toripalimab plus gemcitabine and cisplatin (GP) IC followed by RT (without concurrent cisplatin) with standard GP IC followed by cisplatin‐based CCRT, in patients with T4N1M0 or T1‐4N2‐3M0 NPC. The results showed that the concurrent cisplatin–sparing regimen was non‐inferior to the standard therapy for the primary endpoint of 3‐year failure‐free survival (FFS) (88.3% vs. 87.6%; *p* = 0.002), with no statistically significant differences observed in secondary endpoints such as overall survival (OS). In the superiority analysis for safety, the concurrent cisplatin–sparing group had a significantly lower incidence of all‐grade vomiting than the standard therapy group (26.2% vs. 59.8%; *p* < 0.001). Additionally, grade 3–4 acute adverse events were substantially reduced, and patient‐reported QoL scores were significantly better in the concurrent cisplatin–sparing group [4].

Another single‐arm, phase 2 PLATINUM trial, published by Xu et al., explored the application of nivolumab combined with IC and RT (without concurrent cisplatin) in 152 patients with LANPC [5]. This study also yielded encouraging results, with a 3‐year FFS of 88.5%, which compared favorably with the historical benchmark of 78% in a non‐randomized comparison. Moreover, the incidence of grade 3–4 acute adverse events throughout the treatment period was only 40.2%, indicating a substantially improved tolerability. Notably, the PLATINUM study further explored the predictive value of plasma Epstein–Barr virus deoxyribonucleic acid (EBV DNA) kinetics, finding that patients achieving early clearance of EBV DNA after IC had superior 3‐year FFS compared with those who did not (90.4% vs. 82.9%) [5].

Despite differences in sample size and drug choices, both studies supported omitting concurrent cisplatin chemotherapy in the era. In particular, the findings from the PLATINUM study regarding early EBV DNA clearance suggest that this toxicity‐reducing strategy may have a higher application value in patients who respond well to IC, providing a theoretical basis for future biomarker‐guided, precise de‐escalation therapy. However, it should be emphasized that omitting concurrent cisplatin is currently supported mainly in specific high‐risk populations within endemic regions and requires external validation before wider application.

### 2.2 Efficacy‐Enhancing: Adjuvant Immunotherapy for High‐Risk LANPC

Even with definitive treatment (IC plus CCRT), patients with high‐risk LANPC remain at a significant risk of disease relapse. Before 2025, encouraging evidence had already emerged from the phase 3 CONTINUUM trial. In this study, compared with the standard treatment alone, the sintilimab‐based regimen significantly improved event‐free survival (EFS) in patients with high‐risk LANPC [6]. However, immunotherapy in multiple treatment phases increases treatment complexity, cumulative toxicity, and cost. Therefore, more refined treatment strategies are needed to maximize efficacy while minimizing the treatment burden. The DIPPER study by Liang et al. was the first phase 3 clinical trial to demonstrate that adjuvant PD‐1 blockade can significantly improve outcomes in high‐risk LANPC [3]. This study enrolled high‐risk patients (T4N1M0 or T1‐4N2‐3M0) with no disease progression after IC plus CCRT, and randomized them to receive either adjuvant camrelizumab or observation. The results showed that the adjuvant immunotherapy group achieved a 3‐year EFS rate of 86.9%, which was significantly higher than the 77.3% rate in the observation group (hazard ratio (HR) 0.56, *p* = 0.01). In terms of safety, adjuvant immunotherapy was associated with an increased incidence of low‐grade immune‐related adverse events but did not significantly compromise patients’ QoL. In pre‐specified subgroup analyses, no significant interaction *p* values were observed in any subgroup, indicating a consistent benefit of camrelizumab. For recurrence patterns, the DIPPER trial demonstrated that adjuvant camrelizumab significantly improved both 3‐year distant metastasis‐free survival (92.4% vs. 84.5%; HR, 0.54) and locoregional relapse‐free survival (LRRFS, 92.8% vs. 87.0%; HR, 0.53) compared with standard therapy [3].

Although this study supports the potential role of adjuvant immunotherapy in the management of LANPC, it had several limitations. Because this study employed a fixed regimen of 12 cycles, the optimal duration of adjuvant therapy remained undefined. Moreover, long‐term survival data and cost‐effectiveness assessments are required before this approach can be considered a standard of care.

To clarify the optimal timing of immunotherapy administration, an ongoing multicenter randomized phase 2 trial (ClinicalTrials.gov identifier: NCT06455410) is evaluating neoadjuvant GP chemotherapy combined with the PD‐L1 inhibitor adebrelimab versus GP chemotherapy alone in high‐risk patients with LANPC. Unlike previous studies, this trial focused specifically on the neoadjuvant setting, aiming to determine whether early immune intervention can improve treatment response and long‐term outcomes.

### 2.3 Optimization of Chemotherapy Regimens

Concurrently with the exploration of “efficacy‐enhancing and toxicity‐minimizing” strategies, the optimal timing for chemotherapy intervention (induction vs. adjuvant) has been further clarified. A phase 3 randomized trial published by Guo et al. [7] directly compared IC plus CCRT with CCRT plus adjuvant chemotherapy (AC) in patients with N2‐3 stage NPC. The results showed that although there was no statistically significant difference in the survival benefit between the two groups, the IC group demonstrated a superior distant metastasis control rate compared with the AC group, albeit with a comparatively lower locoregional control rate [7]. These findings provide crucial guidance for individualized chemotherapy regimens based on the patient's risk of local recurrence and distant metastasis.

## 3 Precision and Standardization in Radiotherapy

RT remains the cornerstone of NPC treatment owing to its deep anatomical location and high radiosensitivity. In the era of IMRT, precise target volume delineation is critical for ensuring efficacy and minimizing toxicity. However, traditional RT planning defines the gross tumor volume (GTV) based on the pre‐IC tumor volume, leading to excessively large irradiated fields. Additionally, significant heterogeneity in defining the clinical target volume (CTV) has persisted across centers. With the publication of international guidelines and key clinical trial results, 2025 marks the beginning of “evidence‐based precision” in NPC RT.

**Figure 1:**
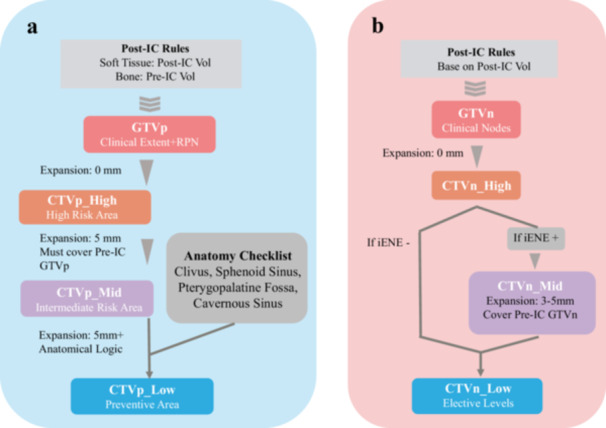
Consensus algorithm for target volume delineation in nasopharyngeal carcinoma (2025 update). (a) Strategy for the primary tumor. The GTVp is defined based on tissue response to induction chemotherapy (IC): soft tissue/mucosal targets are delineated on post‐IC MRI, whereas bone/sinus invasion retains the pre‐IC volume. For cavernous sinus involvement, the pre‐IC extent is included while excluding normal brain parenchyma. CTVp_Low requires specific anatomical inclusion based on T‐stage (e.g., delineating the anterior 1/3 of the clivus for T1‐2 vs. the whole clivus for T3‐4; excluding the sphenoid sinus upper half for T1‐2). (b) Strategy for neck nodes. CTVn_Low covers elective nodal stations, standardly including bilateral levels II, III, Va, and VIIa (lateral RPN), with level Ib included only for high‐risk cases. CTV, clinical target volume; GTV, gross tumor volume; IC, induction chemotherapy; MRI, magnetic resonance imaging; RPN, retropharyngeal nodes.

Regarding the core controversy of “whether GTV should be reduced after induction chemotherapy,” the international guideline led by Tang et al. [8] recommends a post‐IC GTV delineation regimen, in which for soft tissue/mucosal targets (e.g., retropharyngeal and cervical lymph nodes, nasal cavity involvement), delineation should be based on post‐IC magnetic resonance imaging (MRI), while bone/sinus invasion retains the pre‐IC volume (Figure 1A). For cavernous sinus involvement, the pre‐IC extent was included while excluding the normal brain parenchyma [8]. This recommendation is supported by a phase 3 non‐inferiority RCT published by Tang et al. This study enrolled 445 patients who responded to IC and compared “reduced‐field radiotherapy” (planning based on post‐IC GTV) with “conventional radiotherapy” (planning based on pre‐IC GTV). Results demonstrated that the reduced‐field group achieved a 3‐year LRRFS rate of 91.5%, which was non‐inferior to the 91.2% rate in the conventional group. Concurrently, due to the smaller high‐dose irradiated volume, the reduced‐field group had a significantly lower incidence of acute mucositis (19.8% vs. 34.1%), late‐onset otitis media (9.5% vs. 20.9%), and late xerostomia (3.6% vs. 9.5%) [2]. This strategy reflects a growing consensus on using the post‐IC GTV as the delineation benchmark in NPC RT.

Regarding neck target volumes, the concurrently published international guideline similarly incorporates the aim of “precision and toxicity reduction.” Based on big‐data analysis of lymphatic drainage patterns, the new guidelines refined prophylactic irradiation coverage (Figure 1B). The most significant change pertains to the management of level Ib. For patients without clear evidence of metastasis (such as extracapsular extension or lymph nodes > 2 cm), irradiation of level Ib should be omitted [9]. This consensus effectively preserves the submandibular gland function and may help address the long‐standing problem of xerostomia. The guidelines also clarify the anatomical boundaries of each lymphatic drainage region to minimize unnecessary irradiation of normal tissues. These research advancements in 2025 mark a shift in RT for NPC, moving beyond the era of relying on individual experience and entering a new paradigm of “evidence‐based precision.”

Several prospective studies are expected to further refine this precision‐based approach. Notably, an ongoing Multicenter, Non‐Inferiority, Randomized phase 3 trial (ClinicalTrials.gov identifier: NCT06239727) is investigating the impact of reduced‐dose RT in combination with chemotherapy and immunotherapy on patient prognosis, complications, and QoL compared with conventional‐dose RT in combination with chemotherapy and immunotherapy in treatment‐sensitive stage III NPC patients. The results of this study are expected to address an important unanswered question: whether treatment response‐adapted radiotherapy de‐escalation can safely optimize the balance between tumor control and long‐term toxicity.

## 4 Therapeutic Breakthroughs in Recurrent/Metastatic NPC

Before 2025, platinum‐based chemotherapy combined with a PD‐1 inhibitor was the standard first‐line therapy for patients with recurrent or metastatic (R/M) NPC. However, following the failure of first‐line therapy or in patients with platinum‐resistant R/M NPC, traditional salvage chemotherapy options (such as gemcitabine or capecitabine monotherapy) offer limited efficacy. Although early‐stage studies have suggested that immunotherapy‐based combination strategies may overcome PD‐1 resistance, for example, the PD‐1/CTLA‐4 bispecific antibody QL1706 demonstrated encouraging survival benefits in R/M NPC patients [10], a new standard of care has not yet been established. The lack of a standard treatment regimen that significantly prolongs survival highlights the urgent need for novel therapeutic strategies.

Antibody–drug conjugates (ADCs) are a promising therapeutic class that has been investigated in various solid tumors [11]. In patients with R/M NPC progressing after platinum‐based chemotherapy and PD‐1 inhibitor, a phase 3 RCT published by Yang et al. demonstrated the significant efficacy of izalontamab brengitecan (Iza‐bren), an EGFR/HER3‐targeted bispecific ADC for R/M NPC [12]. In this study, the Iza‐bren group achieved a remarkable objective response rate (ORR) of 54.6%, significantly outperforming the chemotherapy group with an ORR of 27.0%. The median progression‐free survival (PFS) was extended to 8.38 months with Iza‐bren compared to only 4.34 months in the chemotherapy group (HR, 0.44) [12]. Although these results suggest that Iza‐bren is a promising candidate for later‐line treatment, mature OS data are required to establish it as a new standard of care. Concurrently, Ma et al. reported the results of a phase I/Ib study of YL201, an ADC targeting B7‐H3. In a cohort of patients with NPC who failed multiple prior lines of therapy, YL201 demonstrated an ORR of 48.6% and disease control rate of 92.9%. Notably, this study found no significant correlation between the efficacy and B7‐H3 expression levels, suggesting the potentially broad applicability of this agent [13]. Together, these two studies confirm that ADCs exhibit promising anti‐tumor activity in patients with R/M NPC.

Addressing the challenge of low response rates to PD‐1 inhibitor monotherapy in later‐line settings, research in 2025 has also provided new strategies through combinations with anti‐angiogenic agents or the introduction of novel immune checkpoint inhibitors. For patients with platinum‐resistant R/M NPC, a phase 2 RCT published by Chong et al. (Singapore) thoroughly investigated the efficacy of pembrolizumab (a PD‐1 inhibitor) combined with bevacizumab (anti‐VEGF). The results showed that the combination group achieved an ORR of 58.3%, which was significantly higher than the ORR of 12.5% in the pembrolizumab monotherapy group (*p* = 0.001). The median PFS was 13.8 months in the combination group versus only 1.6 months in the monotherapy (HR 0.25, *p* < 0.0001) [14]. However, while adding bevacizumab did not increase the incidence of immune toxicities compared with monotherapy, it did result in an increased incidence of grade 3 adverse events, notably stroke and carotid vessel leakage. Furthermore, this study explored the underlying mechanism, suggesting that the VEGF inhibitor remodeled the tumor microenvironment into an immune‐activated state, potentially enhancing the anti‐tumor response. While in the realm of bispecific antibodies, Ma et al. reported long‐term follow‐up data for QL1706, a PD‐1/CTLA‐4 bispecific antibody, showing a median OS of 20.2 months in the NPC cohort. Chen et al. explored the efficacy of an anti‐LAG‐3 antibody in combination with toripalimab. They found that high LAG‐3 expression was associated with a better treatment response, identifying LAG‐3 as a potential target for overcoming PD‐1 resistance [15].

## 5 Artificial Intelligence‐Assisted Diagnosis and Radiotherapy of NPC

In 2025, major advances in deep learning and large language models (LLMs) further expanded the potential applications of AI in NPC management, including diagnosis, RT, and prognosis prediction.

In early screening and diagnosis, AI empowers primary healthcare facilities with expert‐level diagnostic capabilities. Shi et al. developed an STND system based on the Swin Transformer architecture. Validated across multiple national centers, this study achieved a high area under the curve value of 0.99 for identifying NPC during endoscopic examinations [16]. Concurrently, the NPC‐SDNet model proposed by He et al. achieved the segmentation and diagnosis of NPC using multimodal endoscopic data (WLI and NBI images) with a processing speed of 1000 frames per minute, significantly enhancing the clinical screening efficiency [17]. However, both studies have notable limitations in terms of cross‐center reproducibility. Variations in imaging equipment and image acquisition protocols across institutions may lead to discrepancies in image quality, thus impairing model accuracy.

The AI technology has achieved breakthroughs in target delineation and workflow management in RT. The introduction of the Radformer model marked its entry into the vision‐language multimodal approach. This model innovatively employs LLMs to process clinical textual information and achieve language‐aware visual encoding. GTV delineation in head and neck cancers achieved superior accuracy (Dice coefficient of 0.76) compared with traditional vision‐only models (e.g., 3D‐UNETR), significantly reducing boundary delineation errors [18]. Meanwhile, clinical validation of the multistep integrated (MSI) RT workflow confirmed AI's capability for integration. Through seamless AI‐driven auto‐contouring and auto‐planning, the median time from the computed tomography (CT) scan to beam delivery was compressed to 23.2 min, ensuring highly efficient and precise treatment delivery [19].

For efficacy prediction and prognosis assessment, an interpretable graph neural network model based on MRI radiomics not only accurately predicted the response of LANPC to IC but also visually illustrated the rationale behind its decisions, overcoming the trust crisis of traditional “black‐box” models. Furthermore, deep learning‐based digital pathology analysis successfully achieved automated quantification of tumor‐infiltrating lymphocytes from whole‐slide images, providing an objective, independent biological marker for immunotherapy efficacy in NPC [20]. However, in decision‐making clinical scenarios such as treatment recommendations, LLMs face challenges including pre‐training data bias, lack of transparency and interpretability, and outdated knowledge. Retrieval‐augmented generation (RAG), as proposed by Yang et al., may address these issues [21, 22]. By retrieving real‐time evidence from external knowledge sources including clinical guidelines, medical evidence, and patient cases, RAG helps to optimize the output of generative AI models, improving the reliability and transparency of AI outputs to better support clinical judgment.

Collectively, these advances highlight the growing integration of AI into NPC management and its translational potential. Despite promising performance, AI implementation remains limited by the need for external validation, regulatory approval, and broader deployment across diverse clinical settings.

## 6 Advances in Basic and Translational Research

In 2025, basic research on NPC focused on earlier diagnosis, treatment resistance, and novel therapeutic targets with translational potential.

### 6.1 Advances in Diagnosis and Disease Monitoring

The early stages of NPC are often asymptomatic or present with nonspecific symptoms, making early diagnosis challenging. For NPC diagnosis and disease monitoring, Zhang et al. constructed a T‐score model capable of capturing tumor‐specific immune imprints [23]. This model could identify early‐stage NPC 6–12 months before clinical diagnosis, with a sensitivity surpassing that of traditional serological markers, providing a new tool for non‐invasive screening and early intervention in high‐risk populations.

### 6.2 Mechanisms of Treatment Resistance and Identification of Therapeutic Targets

Treatment resistance remains a major obstacle to improving NPC outcomes. You et al. revealed that MCAM+ cancer‐associated fibroblasts in recurrent NPC promote tumor radioresistance through the collagen IV–ITGA2–FAK–AKT signaling axis [24]. This suggests that strategies targeting tumor–stroma interactions may represent a novel direction for overcoming RT resistance. Tang et al. also found that the deubiquitinating enzyme USP5 promotes radioresistance by stabilizing EphA2. Notably, in preclinical models, mebendazole inhibits USP5 expression and exerts radiosensitizing effects; however, these findings require further clinical validation [25].

In the field of chemotherapy resistance, Luo et al. discovered that the common p53 (R280T) mutation induces resistance to 5‐FU by aberrantly activating the DNA repair gene, KMT5B. In preclinical models, the natural agent curcumin downregulates KMT5B expression in NPC cells, thereby restoring chemosensitivity [26]. Yang et al. demonstrated that DDAH1, in a non‐enzymatic manner, directly binds to and activates EGFR, subsequently initiating the JAK2‐STAT3 signaling pathway and inducing cisplatin resistance [27]. Blocking this process with the anti‐EGFR antibody nimotuzumab offers personalized combination therapy for cisplatin‐resistant patients with high DDAH1 expression.

### 6.3 Advances in Novel Immunotherapeutic Strategies

Beyond resistance mechanisms, studies in 2025 further provide new opportunities to enhance the efficacy of immunotherapy. Xie et al. found that the acetyltransferase NAT10 inhibits T‐cell function by upregulating HMGB1 secretion. Inhibition of NAT10 significantly enhanced the efficacy of anti‐PD‐1 therapy, identifying it as a new target for combination immunotherapy [28]. Wang et al. systematically highlighted that the presence of tertiary lymphoid structures has been confirmed in multiple studies to correlate with favorable responses to immunotherapy and that inducing their formation may become a novel strategy to overcome immunotherapy resistance [29].

In summary, the 2025 basic research on NPC has focused on clarifying the mechanisms of treatment resistance and identifying novel therapeutic targets. Integration of these findings supports rational combination strategies, such as targeting the TME with immunotherapy and repurposing agents (mebendazole and curcumin) to block resistance pathways, which show promise for overcoming treatment resistance in NPC.

## 7 Conclusions

The year 2025 marks a significant step forward in the precision and personalization of NPC research and clinical practice. Recent advances have driven progress in NPC treatment across immunotherapy, RT, AI‐assisted diagnosis and treatment, and translational research, improving the therapeutic efficacy and QoL of patients with NPC.

However, several challenges remain to be resolved. Most pivotal studies (e.g., the DIAMOND, DIPPER, and Iza‐bren trials) have been conducted in endemic regions of China and have primarily involved patients with non‐keratinizing carcinoma. Although recent studies indicated that NPC in non‐endemic regions has shifted toward a predominance of World Health Organization type III [30] and is also frequently EBV‐associated [31], efficacy of these strategies in non‐endemic regions and in patients with keratinizing squamous cell carcinoma still requires validation in international multicenter studies. Additionally, the patient populations enrolled in these trials were predominantly fit individuals with an underrepresentation of elderly or comorbid patients noting that caution is warranted when generalizing these findings to unselected real‐world populations. Furthermore, the OS data for agents such as Iza‐bren remain immature, and the long‐term safety and potential late complications of immunotherapy require continued surveillance. In the field of AI, the generalizability of AI models across multiple centers and their integration into real‐world clinical practice are still in progress.

NPC management will continue to evolve toward greater precision, reduced toxicity, and enhanced intelligence. Notably, the shift toward precision medicine depends on the integration of multi‐modal data—including liquid biopsies, radiomics, and digital pathology, thereby enabling dynamic risk monitoring and personalized treatment adaptation. The deep integration of AI into clinical workflows has the potential to restructure care processes and enable intelligent full‐cycle disease management. To translate this potential into clinical practice, future studies should rigorously validate the robustness and generalizability of AI models across diverse clinical settings including different imaging devices, patient populations, and institutional practice patterns. With continued advancements in clinical and basic research, NPC prevention and treatment systems will be increasingly refined, ultimately achieving the paramount goal of enhancing therapeutic efficacy and QoL.

## Author Contributions

**Zishan Liang:** writing – original draft, writing – review and editing, conceptualization. **Xian‐Yue Zeng:** writing – original draft, conceptualization, writing – review and editing. **Jia‐Wei Lv:** conceptualization, writing – original draft, writing – review and editing. **Guan‐Qun Zhou:** conceptualization, writing – original draft, writing – review and editing. **Ying Sun:** conceptualization, writing – original draft, writing – review and editing, supervision. **Li Lin:** conceptualization, writing – review and editing, writing – original draft, supervision.

## Funding

The authors have nothing to report.

## Ethics Statement

The authors have nothing to report.

## Consent

The authors have nothing to report.

## Conflicts of Interest

The authors declare no conflicts of interest.

## Data Availability

Data sharing not applicable to this article as no data sets were generated or analyzed during the current study.
